# Mechanisms of Acetoin Toxicity and Adaptive Responses in an Acetoin-Producing Species, Lactococcus lactis

**DOI:** 10.1128/AEM.01079-21

**Published:** 2021-11-24

**Authors:** Bénédicte Cesselin, Céline Henry, Alexandra Gruss, Karine Gloux, Philippe Gaudu

**Affiliations:** a Université Paris-Saclay, INRAE, AgroParisTech, Micalis Institute, Jouy-en-Josas, France; b Université Paris-Saclay, INRAE, AgroParisTech, PAPPSO, Jouy-en-Josas, France; University of Helsinki

**Keywords:** acetoin, fatty acids, *Lactococcus lactis*, *pst* operon

## Abstract

Acetoin, 3-hydroxyl,2-butanone, is extensively used as a flavor additive in food products. This volatile compound is produced by the dairy bacterium Lactococcus lactis when aerobic respiration is activated by haem addition, and comprises ∼70% of carbohydrate degradation products. Here we investigate the targets of acetoin toxicity, and determine how acetoin impacts L. lactis physiology and survival. Acetoin caused damage to DNA and proteins, which related to reactivity of its keto group. Acetoin stress was reflected in proteome profiles, which revealed changes in lipid metabolic proteins. Acetoin provoked marked changes in fatty acid composition, with massive accumulation of cycC19:0 cyclopropane fatty acid at the expense of its unsaturated C18:1 fatty acid precursor. Deletion of the *cfa* gene, encoding the cycC19:0 synthase, sensitized cells to acetoin stress. Acetoin-resistant transposon mutagenesis revealed a hot spot in the high affinity phosphate transporter operon *pstABCDEF*, which is known to increase resistance to multiple stresses. This work reveals the causes and consequences of acetoin stress on L. lactis, and may facilitate control of lactic acid bacteria production in technological processes.

**IMPORTANCE** Acetoin, 3-hydroxyl,2-butanone, has diverse uses in chemical industry, agriculture, and dairy industries as a volatile compound that generates aromas. In bacteria, it can be produced in high amount by Lactococcus lactis when it grows under aerobic respiration. However, acetoin production can be toxic and detrimental for growth and/or survival. Our results showed that it damages DNA and proteins via its keto group. We also showed that acetoin modifies membrane fatty acid composition with the production of cyclopropane C19:0 fatty acid at the expense of an unsaturated C18:1. We isolated mutants more resistant to acetoin than the wild-type strain. All of them mapped to a single locus *pstABCDEF* operon, suggesting a simple means to limit acetoin toxicity in dairy bacteria and to improve its production.

## INTRODUCTION

**FIG 1:**
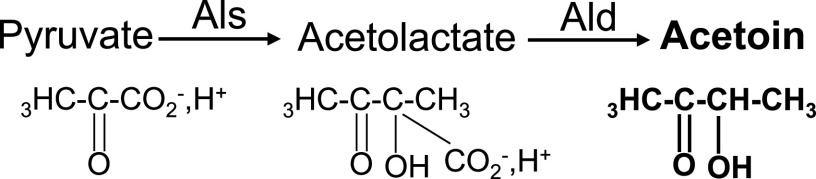
Acetoin biosynthesis pathway in L. lactis MG1363. Als, acetolactate synthase; Ald, acetolactate decarboxylase. Acetoin contains a hydroxyl (R-OH) and a keto (R_1_[C═O]R_2_) group.

Acetoin, also known as 3-hydroxyl,2-butanone, has diverse uses in industry, e.g., for chemical synthesis of heterocyclic compounds, as an e-cigarette ingredient (1), in agriculture as a plant growth factor and inducer of systemic resistance to infection (2, 3), and in dairy industries as a volatile compound that generates aromas (4). Acetoin may be produced chemically or by metabolic engineering. In bacteria, acetoin synthesis involves an acetolactate synthase (Als) to convert pyruvate to acetolactate, and a decarboxylase (Ald) to convert acetolactate to acetoin (Fig. 1).

Lactococcus lactis species, widely used in the food industry, produce mainly lactic acid from carbohydrates via a fermentative metabolism. However, when haem is supplied in the aerated culture, cells trigger a respiratory chain activity that reprograms carbon metabolism (5–7). Rather than producing lactic acid, cells reroute pyruvate to acetate but mainly to acetoin, which represents at least 70% of carbohydrate degradation products (i.e., 35 mM from 55 mM glucose (5–7)). Acetoin production might serve as a release valve to avoid over-accumulation of pyruvic acid, which would block glycolysis. Acetoin helps prevent internal bacterial acidification due to pyruvic acid accumulation when cells are grown in carbohydrate-rich medium (8–10). In L. lactis, its production may be enhanced by metabolic engineering cells that block pyruvate degradation pathways (11, 12). While acetoin production makes metabolic sense, accumulation of acetoin or diacetyl, a derivative of acetoin synthesis pathway, may nevertheless be toxic in bacteria, as high acetoin production was reported to be detrimental for cell growth in L. lactis and *Bacillus* species (11, 13, 14).

Here, we set out to understand the mechanisms and targets of acetoin toxicity, and to assess L. lactis potential responses to acetoin. Our results show that acetoin toxicity relies on its keto group, and has multiple targets. Remarkably, acetoin triggers major changes in membrane fatty acid composition. We isolated acetoin-resistant mutants, which all mapped to a single locus (*pstABCDEF* operon), suggesting a simple means to limit toxicity in dairy bacteria.

## RESULTS

### Acetoin provokes DNA and protein damage.

**FIG 2:**
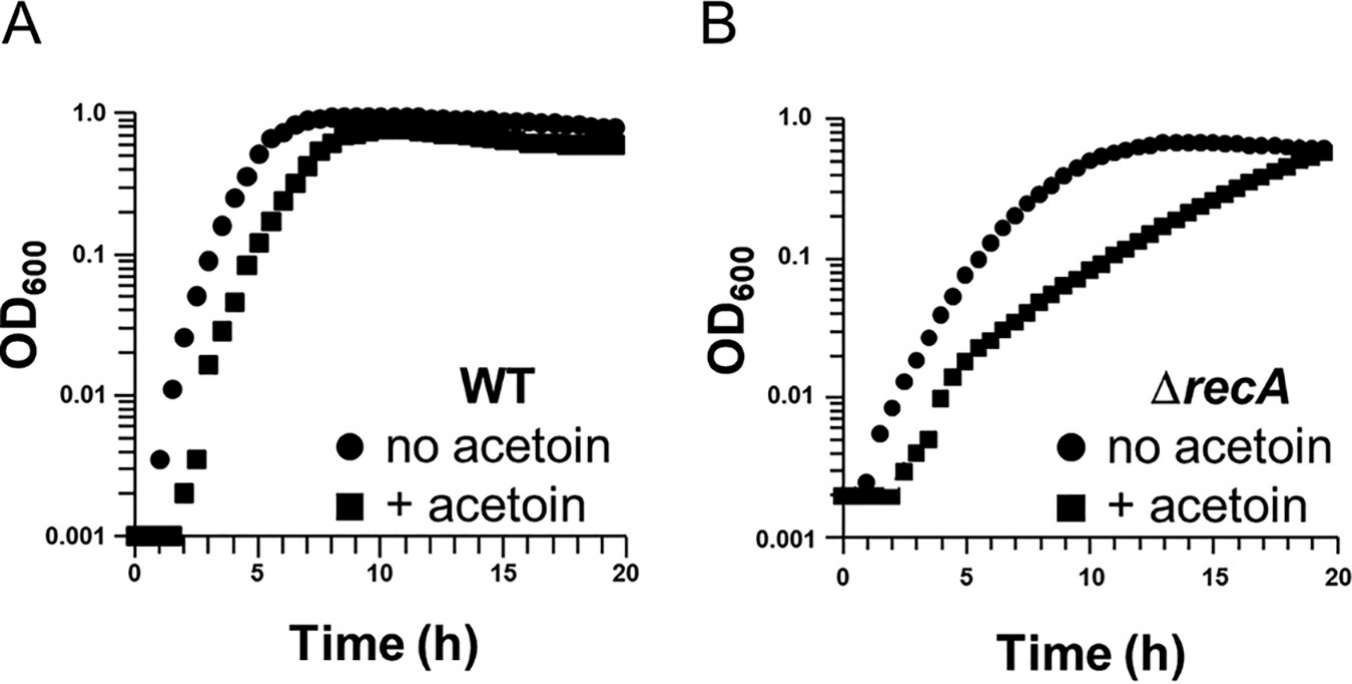
Deletion of *recA* leads to acetoin sensitivity. Growth curves of (A) the WT strain and (B) a Δ*recA* mutant without (circles) and in the presence of (squares) 0.2 M acetoin, under static fermentation in M17Glu1%. Curves are presented in logarithmic scale and representative of three independent experiments.

**FIG 3:**
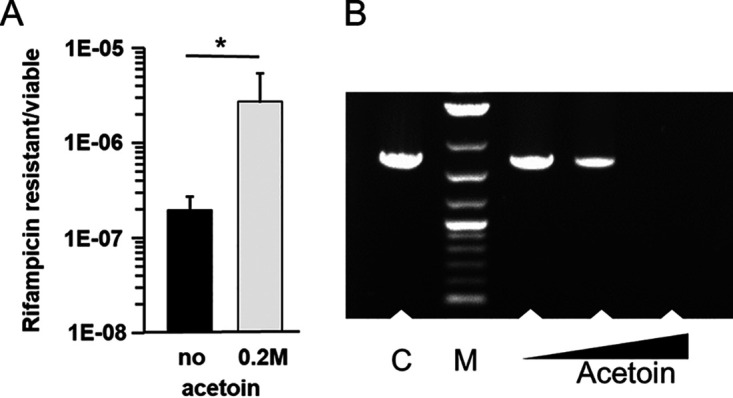
Acetoin is mutagenic and inhibits DNA polymerase. (A) Cells were grown in M17Glu1% under static growth conditions in the presence of 0.2 M acetoin or not. After overnight growth, cultures were diluted and each suspension was spread on solid medium supplemented with rifampin or not. Data, presented in logarithmic scale, are means with standard deviations from three independent experiments. Statistical significance was determined by unpaired, nonparametric Mann-Whitney tests, as recommended for small sample sizes. *, *P*  ≤ 0.05. (B) In a PCR mixture acetoin is added at 11, 22, 33 mM (left to right). C, PCR control with no acetoin; M, 1 log DNA ladder. Photo is representative of two independent experiments.

We tested for toxicity of glucose catabolic products (acetoin, lactate, acetate) on L. lactis strain MG1363 (15) (Table 1). Among them, acetoin had the strongest inhibitory effect (data not shown). In M17Glucose 1% medium, addition of acetoin (70–200 mM) decreased final biomass yields in a dose-dependent manner after either fermentation (static or aerated liquid medium) or respiration growth (aerated liquid medium supplemented with 5 μM haem) (Fig. S1). To understand the mechanisms underlying acetoin toxicity, we first tested its effects on a Δ*recA* mutant. RecA is a main DNA recombination and repair protein that contributes to bacterial protection survival upon DNA damage (16). Compared to growth in non-supplemented medium, addition of 0.2 M acetoin (∼ 6-fold higher of acetoin production under respiration in laboratory M17 broth (4–6)) led to a slight reduction in growth rate of the wild-type (WT) strain compared to a marked reduction of the Δ*recA* strain (Fig. 2); these differences were accentuated upon plating (3-log and 6-log decreases of WT and Δ*recA* strains, respectively; Fig. S2A). We suspected that acetoin is mutagenic. To test this hypothesis, we compared mutation frequencies of the WT strain with and without acetoin using a rifampin assay. The number of rifampin resistant clones was ∼ 15-fold higher in the presence of 0.2 M acetoin than in its absence (Fig. 3A). Acetoin was also found to inhibit DNA synthesis using a PCR assay (Fig. 3B). We observed a net decrease of polymerization efficiency when the enzyme was exposed to physiological concentrations of acetoin (L. lactis synthesized up to 35 mM acetoin in respiration growth from 1% glucose). At 33 mM acetoin we detected no PCR product. As *Taq* polymerase was sensitive to acetoin we tested effects of acetoin on a Δ*clpP* mutant. ClpP is an intracellular housekeeping protease that is required for protein turnover and stability (17). As observed for a Δ*recA* mutant, a Δ*clpP* mutant was more sensitive to 0.2 M acetoin than in the WT strain (Fig. S2B and S3). Altogether, these observations suggest that acetoin leads to mutations and inhibition of protein stability and/or activity including that of DNA polymerase.

**TABLE 1:** Strains, plasmids, and primers

Strain, plasmid, or primer	Description or sequence	Source
Strains		
L. lactis		
MG1363	Plasmid free, L. lactis subsp. *cremoris*	(15)
*pstA*	MG1363, insertion of pRV300 into *pstA* gene	(8)
*pstE::ISS1*	MG1363, insertion element ISS1 into *pstE* gene	This study
Δ*recA*	MG1363, *recA*::Tet^R^	(16)
Δ*clpP*		(17)
*recA*, *pstE::ISS1*		(41)
Δ*clpP*, *pstA*		(8)
Δ*cfa*	MG1363, deletion of *cfa* gene	This study
Δ*cfa*, *pstA*	Disruption of *pstA* gene in a *cfA* mutant	This study
E. coli DH5α	Host strain for cloning	
Plasmids		
pRV300-*pstA*		(8)
pIL252	Ery_L.lactis_, a low-copy-number plasmid	(42)
pGhost9::ISS1	Plasmid for random mutagenesis	(36)
pBR322-pGhost8		(5)
Primers		
ISS1-pEcoR1	TAGTTCATTGATATATCCTCG	
ISS1-pHindIII	GGTATCTACTGAGATTAAGG	
cfa For	TTTTGAATTCCAGTAAGTTTTTCAATGGCG (EcoRI)	
intRev	ACATGTATTCGCGTGTCATTTCGAAAGCAACTGCAAGTGC	
cfa intFor	GCACTTGCAGTTGCTTTCGAAATGACACGCGAATACATGT	
cfa Rev	TTTGGATCCTGGATGCCCTTCTGATACT (BamHI)	
Δcfa extFor	TAACCCATTTGATTTGTGGT	
Δcfa extRev	TGGCATCTGAGATTTGGTCA	

### Acetoin toxicity is based on its keto group.

**FIG 4:**
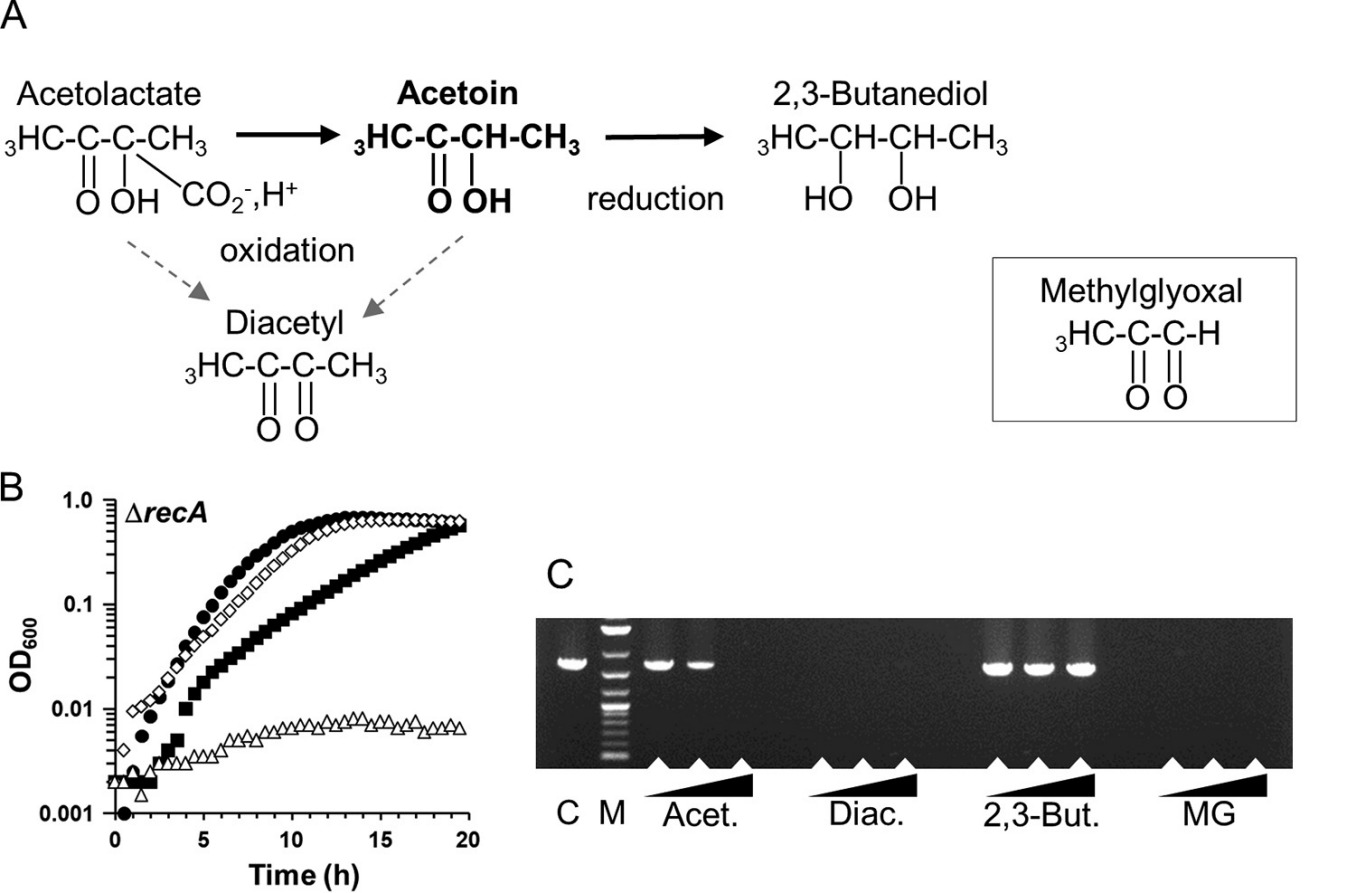
Toxicity of acetoin depends on its keto group. (A) Structure of acetoin and derivatives: diacetyl is produced from spontaneous oxidation of acetolactate or acetoin, whereas 2,3-butanediol by reduction. Structure of diacetyl is similar to methylglyoxal. (B) Growth of a Δ*recA* mutant without and with different compounds: no compound, black circle; acetoin, black square; 2,3-butanediol, diamond; diacetyl, triangle. Cells were cultured in M17Glu1%. Each compound was tested at 0.2 M. Curves are presented in logarithmic scale and representative of three independent experiments. Methylglyoxal gave similar results to that of diacetyl ones. (C) PCR assays. Acet, acetoin; Diac, diacetyl, 2,3-But, 2,3-butanediol; MG, methylglyoxal. Experiments are performed as described in Fig. 3.

To investigate the mechanism of acetoin toxicity, we compared sensitivity of a Δ*recA* mutant to molecules having a similar structure to acetoin: diacetyl, 2,3-butanediol, and methylglyoxal (MG) (Fig. 4A). Like acetoin, diacetyl and 2,3-butanediol are used as flavor additives, whereas MG is a well-known toxic by-product of carbon metabolism in bacteria (18). The reduced form of acetoin (2,3-butanediol) was not toxic, in contrast to its oxidized form (diacetyl), which arrested growth (Fig. 4B). Moreover, diacetyl completely inhibited Δ*recA* strain growth even at very low concentrations, as observed with MG (0.5 mM, data not shown). Using the PCR assay, DNA polymerization efficiency was not affected by the presence of 2,3-butanediol (Fig. 4C). In contrast, no PCR product was detected when the enzyme was exposed to diacetyl or MG. Acetoin treatment led to intermediate effects (Fig. 4B and C). Significant effects of these compounds were also observed in a Δ*clpP* mutant (Fig. S3). Based on these data, we conclude that the keto group is responsible for acetoin toxicity.

### Acetoin treatment affects L. lactis proteomic profiles.

To gain insight on the consequences of acetoin on L. lactis protein expression, we performed proteomic analyses in cells non-treated or treated with acetoin (Table 2, Fig. S4; see Materials and Methods). Compared to the control condition, acetoin treatment resulted in decreased detection of ∼30 proteins belonging to main categories: fatty acid, stress, DNA and translation. No over-produced proteins were detected upon acetoin treatment. The observation that DNA metabolism and translation were affected might be related to acetoin sensitivity of the Δ*recA* and Δ*clpP* mutants and the DNA polymerase. Three fatty acid synthesis (FASII) proteins were decreased: AccC, a subunit of the ACC FASII initiation complex involved in malonyl-CoA synthesis from acetyl-CoA, and FASII elongation enzymes FabF (3-oxoacyl-acyl carrier protein synthase) and FabZ (3-hydroxy-acyl-[acyl-carrier-protein] dehydratase). The decrease of these protein amounts might suggest that acetoin affects membrane fatty acid composition.

**TABLE 2:** Proteins significantly affected by acetoin in L. lactis strain MG1363

Gene name	Protein name/function^*a*^	Expression change in acetoin
	**Glycolysis**	
*llmg_2539*	GapB glyceraldehyde 3-phosphate dehydrogenase	Down
	**Fatty acid biosynthesis**	
*llmg_1779*	AccC subunit biotin carboxylase	Down
*llmg_1783*	FabF 3-oxoacyl-acyl carrier protein synthase II	Down
*llmg_0538*	FabZ (a) 3-hydroxy-acyl-[acyl-carrier-protein] dehydratase	Down
	**Cell wall**	
*llmg_0209*	RmlB, dTDP-glucose-1-phosphate dehydratase	Down
*llmg_0210*	RmlD, dTDP-4-dehydrorhamnose reductase	Down
	**Nucleosides and nucleotides metabolism**	
*llmg_1107*	PyrF, orotidine-phosphate decarboxylase	Down
*llmg_1599*	DeoD, purine nucleoside phosphorylase	Down
*llmg_2176*	Upp, uracyl phosphoribosyltransferase	Down
	**Peptidases**	
*llmg_0403*	PepA, glutamyl aminopeptidase	Down
	**Transcriptional regulators**	
*llmg_0775*	CcpA, catabolite control protein A	Down
	**Cell division**	
*llmg_2060*	FtsZ, cell division protein	Down
	**Stress proteins**	
*llmg_1352*	TelA, putative tellurium resistance protein	Down
*llmg_1996*	Ppa, inorganic pyrophosphatase	Down
*llmg_1498*	iron-sulfur cluster biosynthesis	Down
	**DNA metabolism**	
*llmg_2474*	SsbB, single-strand binding protein	Down
	**Translation**	
*llmg_2373*	RplN, 50S ribosomal protein L14	Down
*llmg_2362*	RplO (a)^*b*^ 50S ribosomal protein L15	Down
*llmg_2362*	RplO (b) 50S ribosomal protein L15	Down
*llmg_0296*	RpsD, 30S ribosomal protein S4	Down
*lLmg_1815 or 2362*	*RplI ribosomal protein or RplO	Down
*llmg_2371 or 2366*	*RplE ribosomal protein or RplF	Down
*llmg_2475*	RpsF (a) 30S ribosomal protein S6	Down
*llmg_2475*	RpsF (b) 30S ribosomal protein S6	Down
*llmg_2557*	RpsG 30S ribosomal protein S7	Down
*llmg_2284*	Frr ribosome recycling factor	Down
*llmg_2429*	Tsf elongation factor	Down
	**Unclassified protein**	
*llmg_0592 or 0296*	*Unknown or RpsD	Down
*llmg_0890 or 0296*	* PyrR, pyrimidine regulator or RpsD	Down
*llmg_2285 or 1599*	*PyrH UMP-kinase or DeoD	Down
*llmg_0763 or 2492*	*Pta phosphate acetyltransferase or Tsf	Down

^*a*^The selected proteins show the most significant differences due to acetoin by visual inspection of duplicate gels (see Fig. 8).

^*b*^(a) and (b) represent the same protein with two different isoelectric points.

*Two proteins with high percentage of coverage were present in one spot.

### Acetoin stress dramatically alters L. lactis fatty acid composition.

**FIG 5:**
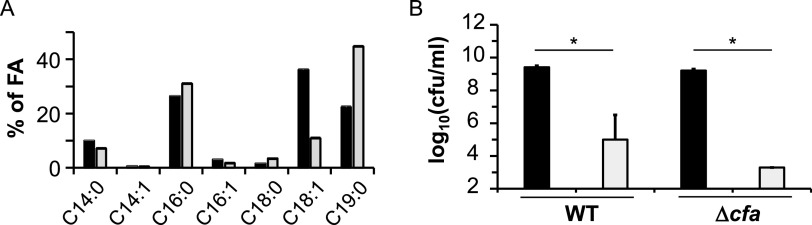
Fatty acid C18:1 cyclopropanation to cycC19:0 participates in acetoin resistance. (A) The WT strain was cultured in M17Glu0.5% at 30°C and collected at OD_600_ = 0.5 for membrane fatty acid extraction and analysis. 0.2 M acetoin was added at OD_600_ = 0.1. Black bars, no acetoin; gray bars, with acetoin. Results are means with standard deviations from three independent experiments. (B) Overnight cultures of the WT strain and a Δ*cfa* mutant were diluted in M17 broth, and 5 μl of each dilution was loaded on agar M17Glu supplemented with 0.35 M acetoin. After 48 h, bacterial counts were determined. Data are means with standard deviations from three independent experiments. Black bars, no acetoin; gray bars, with acetoin. Statistical significance was determined by unpaired, nonparametric Mann-Whitney tests, as recommended for small sample sizes. *, *P* ≤ 0.05.

The observed decrease in FASII enzyme amounts in acetoin-treated cells, and the fact that acetoin is membrane-miscible led us to analyze how this molecule affects membrane fatty acid composition (Table 2). To test this, cells grown without or with 0.2 M acetoin were collected during exponential growth phase (OD_600_ = 0.5), and fatty acids were analyzed by gas chromatography. Non-treated cells produced mainly 4 fatty acids (FAs): myristic acid (C14:0, 10%), palmitic acid (C16:0, 26.5%), oleic acid (C18:1, 36%), and cyclic methylenoctadecenoic acid (cycC19:0, 22%) (Fig. 5A). Addition of acetoin led to a marked increase in the proportion of cycC19:0 FA, to the detriment of its precursor C18:1 FA (2-fold increase and >3-fold decrease, respectively).

CycC19:0 FA is produced from C18:1 FA via the *cfa* gene product, a cyclopropane FA synthase (19, 20). To determine the role of C18:1/cycC19:0 shift in acetoin stress resistance, we compared bacterial survival between the WT strain and a Δ*cfa* mutant treated or not with 0.35 M acetoin (Fig. 5B). These strains grew similarly in the absence of treatment. In contrast, acetoin sensitivity was exacerbated in the *cfa* deletion strain (30-fold greater acetoin sensitivity than the WT strain). We conclude that C18:1/cycC19:0 shift contributes to acetoin resistance in L. lactis.

### The *pst* operon, encoding the high affinity phosphate transporter, is a mutational hot spot for acetoin resistance.

**FIG 6:**
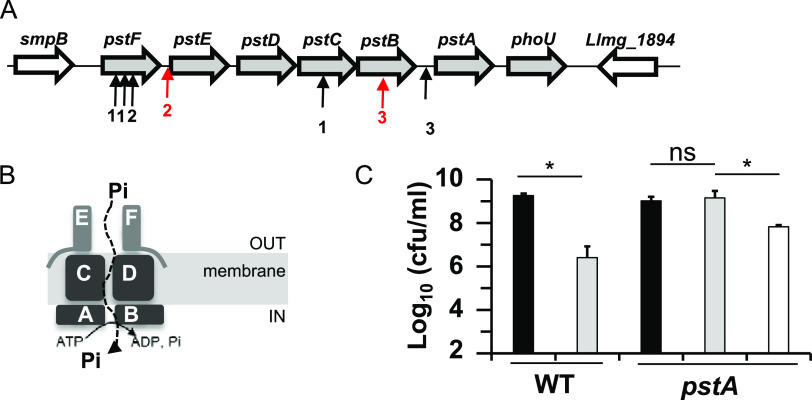
Inactivation of the *pst* operon enhances acetoin resistance. (A) Locus of *pstABCDEF* operon. Arrows indicate insertion site of transposon; red, clones isolated under respiration condition; black, clones isolated under aerobic fermentation condition (B) Schema of phosphate transporter in membrane: PstE (also named PstS) and F, lipoproteins; PstC and D, permease; PstA and B, ATPases; Pi, inorganic phosphate. (C) Resistance of a *pstA* mutant and the WT strain to acetoin. After overnight growth, cells were diluted and 5 μl of each suspension was loaded on M17Glu1% agar plate supplemented with 0.4 M acetoin. Bacterial counts were determined after 48 h of incubation. Data are means with standard deviations from three independent experiments. Black bars, no acetoin; gray bars, with acetoin; white bar, with acetoin and 20 mM phosphate (Pi). Statistical significance was determined by unpaired, nonparametric Mann-Whitney tests, as recommended for small sample sizes *, *P* ≤ 0.05. ns, not significant.

Our results indicated that L. lactis growth and survival may be limited by accrued acetoin production, particularly in respiration conditions where acetoin concentrations reach high levels. One way to overcome toxicity is to select for acetoin-resistant mutants. To this purpose, and to identify functions involved, we conducted transposon mutagenesis in L. lactis strain MG1363 (8). We constructed a mutant library and screened cells growing in the presence of the minimum lethal acetoin concentration, which was determined to be 0.45 M under respiration conditions at 30°C on agar plates (see Materials and Methods). After 48 h of incubation at 30°C, colonies appeared at a frequency of ∼10^−6^. Remarkably, out of 5 selected stable colonies, all transposon insertion sites mapped to the *pstABCDEF* operon (*llmg*_1896 to *llmg*_1901 (15)), which encodes a high affinity phosphate transport complex (Fig. 6A and B) (8). This selection was also performed on L. lactis but growing under aerobic fermentation (agar medium with no haem addition, with a minimum lethal acetoin concentration of 0.65 M). Remarkably, all transposon insertion sites mapped again to the same operon (in 8 mutant colonies tested), indicating that energy mode did not impact the functions giving rise to acetoin resistance. A *pstA* mutant, previously constructed in our lab (8), was ∼100-fold more resistant to acetoin than the WT strain (Fig. 6C). As expected, phosphate supplementation (20 mM) reversed acetoin resistance of the mutant, while it did not affect the WT strain (data not shown).

**FIG 7:**
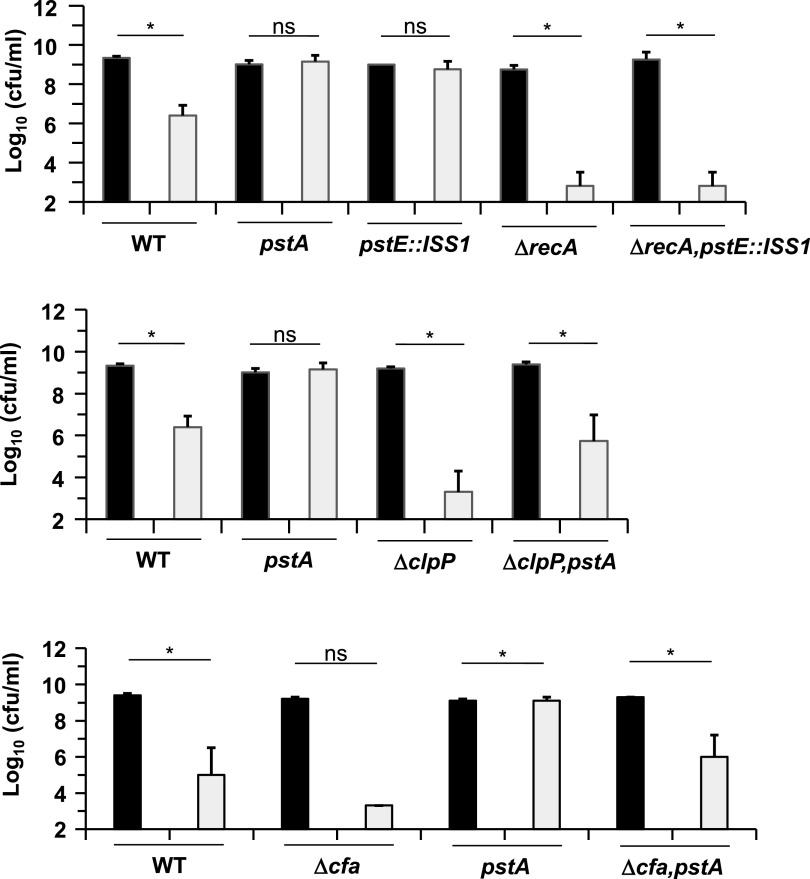
The *pst* operon deletion partially rescues Δ*clpP* and Δ*cfa* mutants against acetoin toxicity but not a Δ*recA* mutant. Overnight cultures were tested on M17Glu agar plates as described in Fig. 6. Data are means with standard deviations from three independent experiments. Acetoin was added at 0.35 M in plates. Black bars, no acetoin; gray bars, with acetoin. Statistical significance was determined by unpaired, nonparametric Mann-Whitney tests, as recommended for small sample sizes, *, *P* ≤ 0.05; ns, not significant.

We determined above that acetoin affected *L lactis* on three levels: DNA damage repair, protein turnover, and membrane lipid function, seen respectively as growth sensitivity of the Δ*recA*, Δ*clpP.* and Δ*cfa* mutants (Fig. 2, Fig. S2 and S5). We asked whether *pst* inactivation rescues survival to an acetoin challenge by constructing the respective double mutants (*pstE-*Δ*recA*, *pstA-*Δ*clpP*, or *pstA-*Δ*cfa*). The *pstA* mutation partially rescued both Δ*clpP* and Δ*cfa* mutants from acetoin toxicity; however, it did not rescue the Δ*recA* strain (Fig. 7).

**FIG 8:**
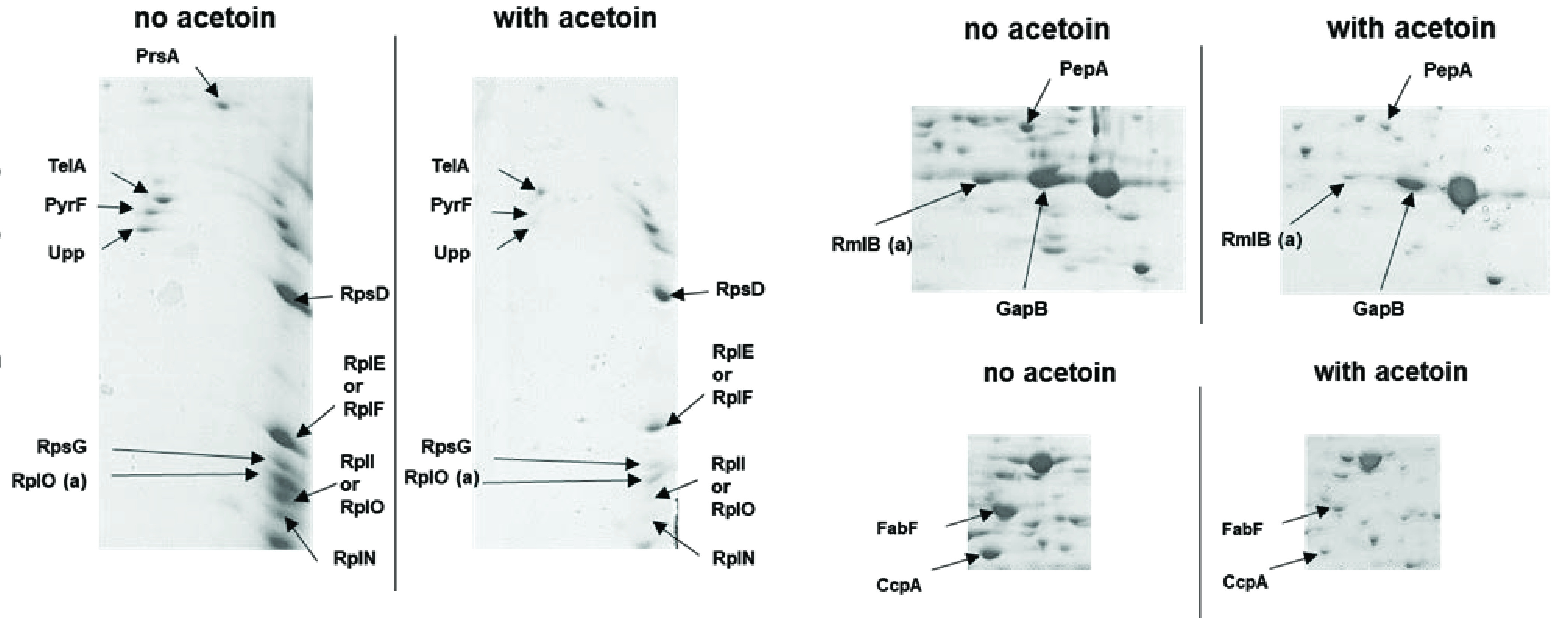
Acetoin affects the proteome of L. lactis strain MG1363. The WT strain was treated or not to 0.2 M acetoin. Cytosolic proteins were separated according to their isoelectric point (first dimension) then according to their molecular weight (second dimension). Proteins were stained by InstantBlue dye. Identification of proteins was determined from mass spectrometry analysis. Figure is representative of two independent experiments (see Materials and Methods). Phosphoproteome analysis is described in the supplemental material.

To obtain insight into acetoin resistance in the *pstA* mutant we performed a comparative (phospho)-proteome analysis of the WT and *pstA* strains treated or not with acetoin (Table 2, Fig. 8, Fig. S4, and Tables S1, S2, and S3). Although we observed differences in some protein amounts and phosphorylation states between the two strains, we failed to identify specific proteins or metabolic pathways that would explain acetoin resistance in the *pstA* mutant. Furthermore, fatty acid analysis showed that the *pstA* mutant behaved like the WT strain in response to acetoin stress (Fig. S5). These results suggest that *pstA* rescue affects cell physiology via mechanisms that may not directly affect the interactions of acetoin with its targets. This conclusion is supported by the fact that *pst* mutations arose in independent selections, including dithiothreitol, acidification, and tellurite stress, or in a DNA repair defective strain, combined with high temperature (8, 21, 22).

## DISCUSSION

L. lactis is widely used in dairy industries for its capacity to acidify medium (milk) by fermentation. In contrast, when oxygen and haem are available, it uses respiration metabolism, and produces flavor-enhancing compounds like acetoin and diacetyl. Acetoin biosynthesis pathway avoids accumulation of pyruvate (end product of glycolysis) and thus extends respiration growth (5). However, we show that its beneficial effect can be hindered when acetoin or/and diacetyl is accumulated at high levels. We demonstrate that these molecules cause L. lactis cell damage to DNA and proteins, and has major effects on membrane lipid composition when cells are exposed to high acetoin levels.

Although used for food flavoring, acetoin is nevertheless toxic, and diacetyl, an acetoin oxidation product, is even more toxic. We demonstrate that acetoin reactivity depends on its keto group as its reduction to 2,3-butanediol restored growth of the WT strain and mutants (Δ*recA* and Δ*clpP* mutants), and alleviated inhibition of DNA polymerization *in vitro*. Moreover, the presence of two close keto groups enhances reactivity of molecules, as evidenced by greater toxicity of diacetyl and methylglyoxal (MG) compared to acetoin. MG damages macromolecules like DNA and proteins in E. coli or B. subtilis and is also proposed to be a source of human pathologies (23). Concerning proteins, acetoin could react preferentially with guanidino rather than amino groups of amino acid residue as reported for diacetyl. *In vitro*, diacetyl reacts with the arginine residue (Arg-235) of Lactobacillus plantarum d-lactate dehydrogenase, as its substitution by a lysine residue enhanced enzyme resistance to diacetyl (24). Our observations likely explain the respiratory pathologies observed in humans exposed to diacetyl and acetoin during production or in food products. Accordingly, previous investigations in animal models reported that a 6-h inhalation exposure to diacetyl caused epithelial damage (25, 26).

This study raises the question of how L. lactis cells cope with acetoin/diacetyl stress. In E. coli, systems like glyoxalase/deglycase that repair damage due to MG have been characterized (23), but such a system is still unknown in L. lactis. Another way should be the reduction of acetoin and diacetyl to 2,3-butanediol, which is inert with respect to growth or DNA polymerization. Previous transcriptome analyses under respiration revealed overproduction of potential NADH reductases (*butA* and *butB* genes) in L. lactis strains having high similarity to acetoin reductase (4, 5, 27). While these functions might be involved in acetoin detoxification, cloning of *butA*, *butB*, and *butAB* from L. lactis strain MG1363 on a multicopy plasmid had no effect on acetoin degradation (unpublished data); it remains to be tested whether these proteins require some limiting cofactors, or if the right conditions for their activities were not found. Nevertheless, variable expression of these potential detoxifying enzymes might explain a reported failure to detect acetoin/diacetyl reductase activity in L. lactis strain MG1363, although the corresponding genes are present (11). The absence of this activity may compromise survival of cells exposed to these molecules.

An unexpected finding is that acetoin addition resulted in a marked change in L. lactis fatty acid membrane phospholipid profiles. The C18:1/cycC19:0 shift likely reflects an increased production or activity of Cfa (28). A *cfa* deletion led to extreme acetoin sensitivity, indicating that Cfa confers efficient protection against acetoin toxicity. In Bacillus amyloliquefaciens, an acetoin resistant mutant displayed increased carbon chain unsaturation, with reported production of C18:1, C18:2, and C18:3 FAs (13). Although *Bacillus* species do not synthesize unsaturated fatty acids, their desaturase activity, possibly increased in the reported *B. amyloliquefaciens* acetoin-resistant mutant, could generate unsaturated species (29, 30). The production of unsaturated FA, and in the present study, cyclopropane FA, may help stabilize membrane fluidity in contact to acetoin (31).

We performed an unbiased mutagenesis to characterize factors implicated in acetoin adaptation. Intriguingly, all isolated mutants were affected in phosphate transport. A recent study in E. coli reported that cells escaped methylglyoxal toxicity through mutations in genes involved in phosphate homeostasis including a mutation in phosphate transporter operon (*pstA* gene) (32). This study, and our work, suggests the existence of a link between phosphate homeostasis and resistance against these molecules (acetoin, diacetyl, and MG) in bacteria. Interestingly, the *pst* operon was previously implicated in metal homeostasis in L. lactis, Saccharomyces cerevisiae, and E. coli (8, 33–35). Decreasing the intracellular metal pool might lower the chances of oxidizing acetoin to the more toxic diacetyl.

In conclusion, this study demonstrates that acetoin and diacetyl are toxic to L. lactis and likely to other bacteria and eukaryotic cells. We identify C18:0/cycC19:0 shift as a defense mechanism against acetoin stress. Isolation of acetoin-resistant *pst* mutants might provide a GMO-free means of isolating lactococci for more robust starter culture production whatever the growth mode used. Indeed, not all lactic acid bacteria are capable of respiration metabolism (4).

## MATERIALS AND METHODS

### Strains and growth conditions.

Strains and plasmids are described in Table 1. L. lactis strains were grown in reconstituted M17 broth supplemented with 0.5 or 1% glucose and riboflavin 5 μM, referred to as M17Glucose. Fresh medium was inoculated at OD_600_ = 0.025 from overnight precultures, and cells were grown under static, aeration (shaking, 200 rpm), or respiration (shaking, 200 rpm; 5 μM haem in broth) conditions. Cultures were usually incubated at 30°C and harvested at the indicated cell densities (OD_600_). For assays of acetoin sensitivity, acetoin at the indicated concentrations is added with inoculum and growths were performed in microplates. Cell densities were measured using a plate reader (Sunrise, TECAN), or otherwise as specified in text. When appropriate, erythromycin (Ery) was added at 1 μg ml^−1^.

### Rifampin assays.

Cells were grown in liquid M17Glu1% in the presence of 0.2 M acetoin or not. After overnight growth, cultures were diluted in M17 broth and 0.1 ml of each dilution was spread on solid growth medium supplemented or not with 50 μg ml^−1^ rifampin. Bacterial counts were determined after 48 h of incubation.

### PCR assay.

A fragment (1.7 kb of size) was PCR amplified with primer pairs: for 5’CCGGAATTCTGGTTCGCTTCAATTGATCG3’, Rev 5’CCGCTCGAGTAATCTAAAGACCATTATACC3’, and L. lactis MG1363 chromosome as a matrix in a reaction mixture containing 1X buffer, 0.25 mM each dNTP, 1 μM each primer, and 1 unit of Phusion DNA polymerase (New England Biolabs). PCR was run for 20 cycles.

### Acetoin^R^ mutant isolation.

Insertional mutagenesis was performed using MG1363 carrying the thermosensitive plasmid pGhost9::ISS1 as described (36). Cell dilutions were plated on M17Glu agar plates containing Ery 1 μg ml^−1^ and incubated at 37°C for 48 h. Clones (about 10,000) were scraped from plates and resuspended in M17 medium with glycerol 15% and stocked at –80°C. The cells were screened on plates on M17Glu containing the minimum lethal concentration of acetoin, (defined as no cell growth for 72 h): 0.65 M acetoin in aerobic fermentation conditions, and 0.45 M acetoin in respiration permissive conditions in medium containing 10 μM haem. Plates were incubated at 30°C. Colonies that appeared at 24 and 48 h were streaked on M17Glu plus acetoin 0.45 or 0.65 M for fermentation or respiration growth and incubated at 30°C before identification of ISS1 transposon insertion sites.

### Characterization of transposon targets.

Mutant chromosomes were purified, digested by SspI, and ligated by T4 ligase. The circulated products containing ISS1 transposon were PCR amplified using primers (ISS1-pEcoRI, ISS1-pHindIII, Table 1) and sequenced (Eurofins & GATC society). Sequencing results were blasted against the genome of L. lactis MG1363 (37) to identify transposon insertion site.

### (Phospho)proteome.

### (i) Sample preparation.

Overnight cultures of L. lactis strains were diluted 1/100 in fresh M17Glu0.5% broth in static conditions. At OD_600_ = 0.1, acetoin 0.2 M was added. At OD_600_ = 0.5, cells were harvested by centrifugation at 4°C, and resuspended in 30 mM Tris pH 7, DTT 1 mM. Cells were disrupted with glass beads (FastPrep FP120; MP Biomedical) by three cycles of 45 sec at a speed of 6 m s^−1^. Extracts were centrifuged at 8,000 rpm for 20 min at 4°C. Crude extracts were ultracentrifuged for 1 h at 10°C to separate membrane and cytosolic fractions. Protein concentrations were measured according to the Bradford procedure with bovine serum albumin as the standard (Bio-Rad, France).

### (ii) 2D-Electrophoresis.

Five hundred μg cytosolic protein samples were used for analyses. They were treated with nuclease (purified NucA from Streptococcus agalactiae (38)) for 30 min at 37°C, and 2D gels were performed as described (39). Phosphoproteins were stained by Pro-Q Diamond dye (Invitrogen, France), which detects P-tyrosine, P-serine, and P-threonine, and visualized with Chemidoc MP Imaging System (Bio-Rad, France) with a UV standard filter. Gels were then washed twice in water (20 min, at room temperature) and stained by Instantblue dye (Expedeon, United Kingdom). Two independent experiments were performed. Spots selected by visual inspection were excised from gels for mass spectrometry identification (see the supplemental material).

### Fatty acid analysis.

Fatty acid extractions were performed on cells grown to OD_600_ = 0.5 ± 0.05. Acetoin (0.2 M) was added when indicated at OD_600_ = 0.1. Membrane fatty acid extraction and analysis were performed as described (40).

### Construction of a Δ*cfa* mutant.

We proceeded as follows. Two DNA fragments covering the upstream (cfa For × cfa intRev) and downstream (cfa intFor × cfa Rev) regions of the *cfa* gene (Table 1) were PCR-amplified and then fused by a second PCR. The resulting fragment was ligated to pBR322-pGhost8 into EcoRI and BamHI sites. The modified plasmid was established in E. coli strain DH5α and transferred to L. lactis strain MG1363. Deletion by temperature shift was performed as described (8) and checked by PCR with primer pair outside of *cfa* locus.
