# Supplementary material for: Mechanisms of Acetoin Toxicity and Adaptive Responses in an Acetoin-Producing Species, Lactococcus lactis
Source: Appl Environ Microbiol. 2021 Nov 24;87(24):e01079-21. doi: 10.1128/AEM.01079-21 (PMC8612267; doi:10.1128/AEM.01079-21)
Supplement: Supplemental file 1 — Supplemental text, Tables S1 to S3, Fig. S1 to S5. Download aem.01079-21-s0001.pdf, PDF file, 2.0 MB [file aem.01079-21-s0001.pdf]

AEM01079-21 manuscript R1 version, Supplementary data

Mechanisms of acetoin toxicity and adaptive responses in an acetoin-producing species, *Lactococcus lactis*.

Bénédicte Cesselin[1], Céline Henry[1,2], Alexandra Gruss[1], Karine Gloux[1], and Philippe Gaudu[1]#

[1] Université Paris-Saclay, INRAE, AgroParisTech, Micalis Institute, 78350, Jouy-en-Josas, France

[2] Université Paris-Saclay, INRAE, AgroParisTech, PAPPSO, 78350, Jouy-en-Josas, France.

Running Head: Mechanism of acetoin toxicity in *Lactococcus lactis*

#Address correspondence to Philippe Gaudu, philippe.gaudu@jouy.inra.fr.

E-mail: Tel: (+33) 01 34 65 20 80; Fax: (+33) 01 34 65 20 65.

Université Paris-Saclay, INRAE, AgroParisTech, Micalis Institute, 78350 Jouy-en-Josas, France.

Key words: acetoin, fatty acid, *pst* operon, *Lactococcus lactis*.

Mass Spectrometry

(i) Sample preparation. Gel pieces were destained using Solvent A (10% v/v acetic acid, 40% v/v ethanol) and then Solvent B (50% v/v 50 mM ammonium bicarbonate, 50% v/v acetonitrile). Gel samples were then treated with 10 mM dithiothreitol (Sigma) to reduce proteins, and 55 mM iodoacetamide (Sigma) to alkylate cysteine residues in proteins, followed by digestion with 100 ng trypsin (Promega). Peptides were then extracted using a solution of 0.5 % v/v trifluoroacetic acid and 50 % v/v acetonitrile. They were dried completely using a concentrator (Savant™ SPD121D, Thermo Fisher Scientific) and resuspended in 20 µl loading buffer (0.08% v/v trifluoroacetic acid, 2% v/v acetonitrile) for LC-MS/MS proteome analysis.
(ii) Mass Spectrometry. Mass spectrometry was performed on the PAPPSO platform (MICALIS, INRA, Jouy-en-Josas, France; http://pappso.inra.fr/), using an Orbitrap Fusion™ Lumos™ Tribrid™ (Thermo Fisher Scientific) coupled to an UltiMate™ 3000 RSLCnano System (Thermo Fisher Scientific). A 4 µl treated sample was loaded at 20 µl min^{-1} on a precolumn (µ-Precolumn, 300 µm i.d x 5 mm, C18 PepMap100, 5 µm, 100 Å, ThermoFisher) and washed with loading buffer. After 3 min, the precolumn cartridge was connected to the separating column (Acclaim PepMap®, 75 µm x 500 mm, C18, 3 µm, 100 Å, Thermo Fisher Scientific). Buffer A consisted of 0.1% formic acid in 2% acetonitrile and buffer B of 0.1% formic acid in 80% acetonitrile. The runs were executed at 300 nl min^{-1} with a linear gradient from 0 to 35 % buffer B for 50 min then 55% for 5 minutes. Including regeneration (98% buffer B), one run took 65 min. Ionization (2.8kV ionization potential) and capillary transfer (275°C) were performed with a liquid junction and a capillary probe. Peptide ions were analyzed using Xcalibur 4.1 with the following machine setup in HCD mode: 1) full MS scan in Orbitrap (scan range [m/z] = 400–1800) and 2) MS/MS using HCD (30 % collision energy) in Orbitrap (AGC target = 5.0×10^4, max. injection time = 100 ms, data type = centroid). Analyzed charge states were set to 2-3, the dynamic exclusion to 60 s and the intensity threshold was fixed at 2.0×10^4.

(iii) Data Analyses. The *L. lactis* MG1363 database (UniprotKB, version 2019, 2702 entries) was searched using X!TandemPipeline version 0.2.38 ((1), http://pappso.inra.fr/bioinfo/xtandempipeline/). Protein identification was performed as described: run with a precursor mass tolerance of 10 ppm and a fragment mass tolerance of 10 ppm. Enzymatic cleavage rules were set to trypsin digestion (after Arg and Lys, unless Pro follows directly after) and no semi-enzymatic cleavage rules were allowed. The fix modification was set to cysteine carbamidomethylation and methionine oxidation and S/T/Y phosphorylation were considered as potential modifications. The identified proteins were filtered as follows: 1) peptide E-value $<10^{-2}$ with a minimum of 2 peptides per protein and 2) a protein E-value of $<10^{-4}$. For the phosphoprotein identification: The identified proteins were filtered as follows: 1) peptide E-value $<10^{-3}$ with a minimum of 1 peptide per protein and 2) a protein E-value of $<10^{-2}$. Proteins presented in tables X and Y were identified by at least 40% coverage.

Table S1: Effects of acetoin on proteome of the *L. lactis* strain MG1363 and a *pstA* mutant.

**Protein**/ gene		**Function** / protein name	**WT acetoin/ WT**	***pstA*⁻ acetoin/ *pstA*⁻**
		**Glycolysis**		
**GapB**	*llmg_2539*	glyceraldehyde 3-phosphate dehydrogenas	down	down
		**fatty acid biosynthesis**		
**AccC**	*llmg_1779*	biotin carboxylase	down	down
**FabF**	*llmg_1783*	3-oxoacyl-acyl carrier protein synthase II	down	down
**FabG1**	*llmg_1784*	3-oxoacyl-acyl carrier protein reductase		down
**FabZ (a)**	*llmg_0538*	3-hydroxy-acyl-[acyl-carrier-protein] dehydratase	down	down

		**cell wall**		
**RmlB (a)**	*llmg_0209*	dTDP-glucose-1-phosphate dehydratase	down	down
**RmlD**	*llmg_0210*	dTDP-4-dehydrorhamnose reductase	down	
		**nucleosides and nucleotides metabolism**		
**PyrF**	*llmg_1107*	orotidine-phosphate decarboxylase	down	down
**DeoD**	*llmg1599*	purine nucleoside phosphorylase	down	down
**Upp**	*llmg_2176*	uracyl phosphoribosyltransferase	down	down
**PrsA**	*llmg_1743*	ribose-phosphate pyrophosphokinase		down
		**Peptidases**		
**PepA**	*llmg_0403*	glutamyl aminopeptidase	down	
		**transcriptional regulators**		
**CcpA**	*llmg_0775*	catabolite control protein A	down	down
		**cell division**		
**FtsZ**	*llmg_2060*	cell division protein FtsZ	down	
		**stress proteins**		
**TelA**	*llmg_1352*	putative tellurium resistance protein	down	
**Ppa**	*llmg_1996*	inorganic pyrophosphatase	down	down
	llmg_1498	iron-sufur cluster biosynthesis	down	
		**DNA metabolism**		
**SsbB**	*llmg_2474*	single-strand binding protein	down	down
		**Translation**		
**RplA**	*llmg_2276*	50S ribosomal protein L1		down
**RplN**	*llmg_2373*	50S ribosomal protein L14	down	down
**RplO (a)**	*llmg_2362*	50S ribosomal protein L15	down	down
**RplO (b)**	*llmg_2362*	50S ribosomal protein L15	down	
**RpsD**	*llmg_0296*	30S ribosomal protein S4	down	down
**RpsF (a)**	*llmg_2475*	30S ribosomal protein S6	down	down
**RpsF (b)**	*llmg_2475*	30S ribosomal protein S6	down	down

**RpsG**	*llmg_2557*	30S ribosomal protein S7	down	down
**Frr**	*llmg_2284*	ribosome recycling factor	down	down
**Tsf**	*llmg_2429*	elongation factor Ts	down	
		**Unknown/unidentified protein**		
	llmg_0794	Hypothetic_protein		down
**RplE or RplF**	*llmg_2371 or 2366*	ribosomal protein	down	down
**RplI or RplO**	*llmg_1815* or *2362*	ribosomal protein	down	down
**Llmg_0592 or RpsD**	*llmg_0592* or *0296*	unknown or ribosomal protein	down	down
**PyrR or RpsD**	*llmg_0890* or *0296*	pyrimidine operon regulator or ribosomal protein	down	down
**PyrH or DeoD**		UMP-kinase or purine nucleoside phosphatase	down	down
**Pta or Tsf**		phosphate acetyltransferase or elongation factor	down	down

Table S2: Effects of acetoin on phosphoproteome of the *L. lactis* strain MG1363 and a *pstA* mutant.

**Protein** / Gene		**Function** / Protein name	**WT acetoin / WT**	***pstA⁻* acetoin / *pstA***
		**glycolysis**		
**Pfk**	*llmg_1118*	6-phosphofructokinase		down
**Pyk**	*llmg_1119*	pyruvate kinase	down	
**BglA**	*llmg_0441*	6-phospho-beta-glucosidase	up	
		**amino acids metabolism**		
**MetK**	*llmg_2160*	S-adenosylmethionine synthetase		down
		**fatty acid biosynthesis**		
**FabZ (b)**	*llmg_1781*	3-hydroxy-acyl-[acyl-carrier-protein] dehydratase		down
		**cell wall**		
**RmlB (b)**	*llmg_0209*	dTDP-glucose-1-phosphate dehydratase		down
		**transport system components**		
**PtsI**	*llmg_0127*	phosphoenolpyruvate-protein-phosphotransferase	down	down
		**stress proteins**		
**SodA**	*llmg_0429*	superoxide dismutase	up	up
**DnaK**	*llmg_1574*	dnaK protein	down	down
	llmg_1498	iron-sufur cluster biosynthesis	down	down
		**translation**		
**RplL (a)**	*llmg_1208*	50S ribosomal protein L7/L12	down	down
**RplO (b)**	*llmg_2362*	50S ribosomal protein L15	up	
**Tsf**	*llmg_2429*	elongation factor Ts		down

Table S3: Effect of the *pstA* mutation on phosphoproteome in *L. lactis* strain MG1363

**Protein** / gene		**Function** / Protein name	***pstA* / WT**
		**glycolysis**	
**GapB**	*llmg_2539*	glyceraldehyde 3-phosphate dehydrogenase	up
**Pfk**	*llmg_1118*	6-phosphofructokinase	up
		**fatty acid biosynthesis**	
**AccC**	*Llmg_1779*	biotin carboxylase	up
**FabZ (b)**	*llmg_1781*	3-hydroxy-acyl-[acyl-carrier-protein] dehydratase	up
		**nucleosides and nucleotides metabolism**	
**PrsA**	*llmg_1743*	ribose-phosphate pyrophosphokinase	up
		**DNA metabolism**	
**SsbB**	*llmg_2474*	single-strand binding protein	up
		**translation**	
**RplO (a)**	*llmg_2362*	50S ribosomal protein L15	up
**RpsD**	*llmg_0296*	30S ribosomal protein S4	up
**RpsF (a)**	*llmg_2475*	30S ribosomal protein S6	up

REFERENCES

1. **Langella O, Valot B, Balliau T, Blein-Nicolas M, Bonhomme L, Zivy M.** 2017. X!TandemPipeline: A tool to manage sequence redundancy for protein inference and phosphosite identification. J Proteome Res **16:**494-503.

Figure and Legends

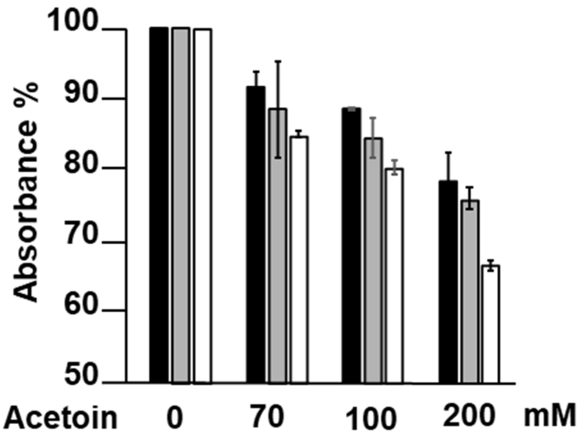

FIG S1 Sensitivity of the *L. lactis* strain MG1363 to acetoin. Cells were cultured in M17Glu1% under static (black bars), aeration (grey bars), and respiration (white bars) growth conditions. After overnight growth in the absence of acetoin, OD_{600} were determined. Data are represented in percentage of growth in the absence of acetoin and represent means with standard deviations from three independent experiments. From overnight growth, OD_{600} measured were 2.7 +/- 0.05; 3.3 +/- 0.5; 5 +/- 0.6 in static, aeration, and respiration condition respectively.

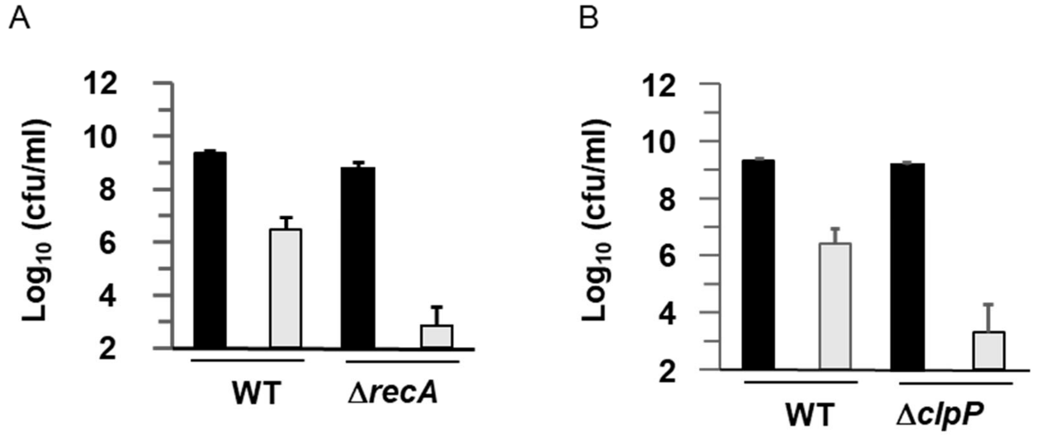

FIG S2 Deletion of *recA* or *clpP* gene enhances acetoin sensitivity in cells. After overnight growth, cells are diluted in M17 broth and 5 µl of each suspension was loaded on M17Glu1% agar plate (black bars) or supplemented with 0.35 M acetoin (grey bars). Bacterial counts were measured after 2 days of incubation. Data, represented with standard deviations, were a mean of three independent experiments.

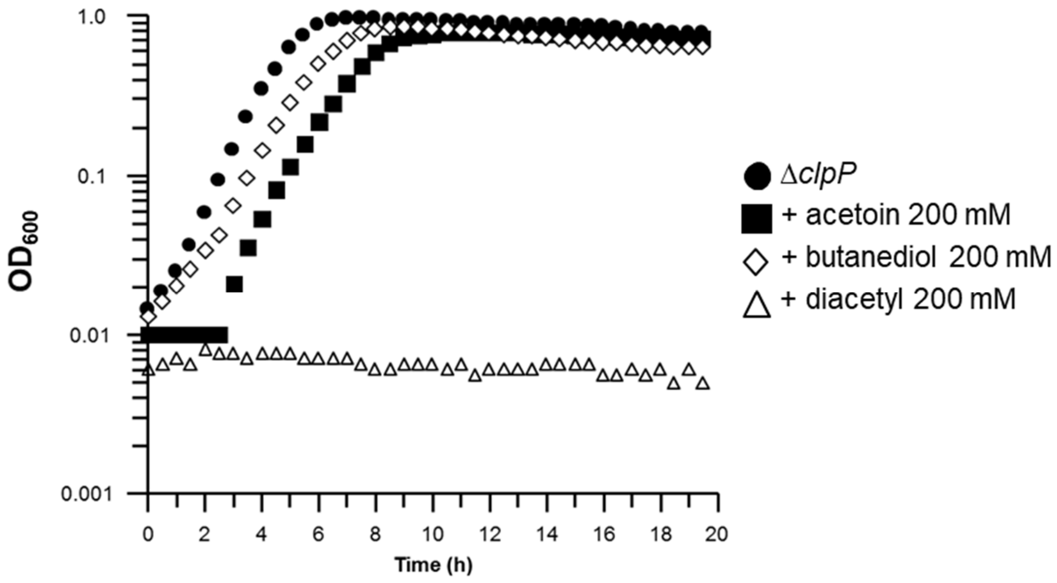

FIG S3. Sensitivity of a Δ*clpP* mutant to acetoin and similar compounds. Cells were cultured under static fermentation in M17Glu1% with 0.2M of compounds: no product (black circle), acetoin (black square); 2,3-butanediol (white diamond); diacetyl or methylglyoxal (white triangle). The cell densities were measured using a plate reader (Sunrise, TECAN). Growth curves are representative of three independent experiments.

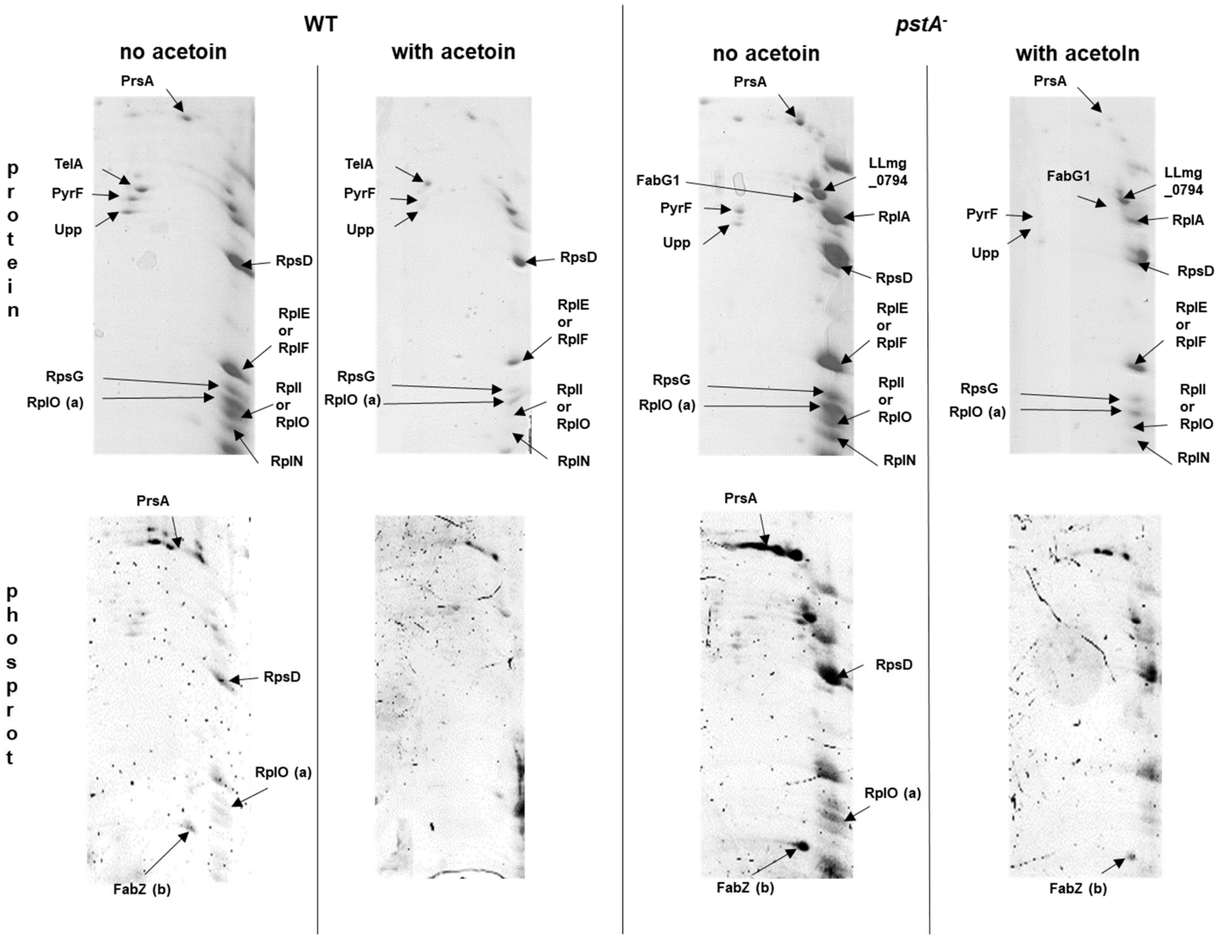

WT
pstA-
no acetoin
with acetoin
no acetoin
with acetoIn
p r o t e i n
p h o s p r o t
PrsA
TelA
PyrF
Upp
RpsD
RplE or RplF
RpsG
RplO (a)
RplI or RplO
RplN
FabG1
LLmg _0794
RplA
FabZ (b)

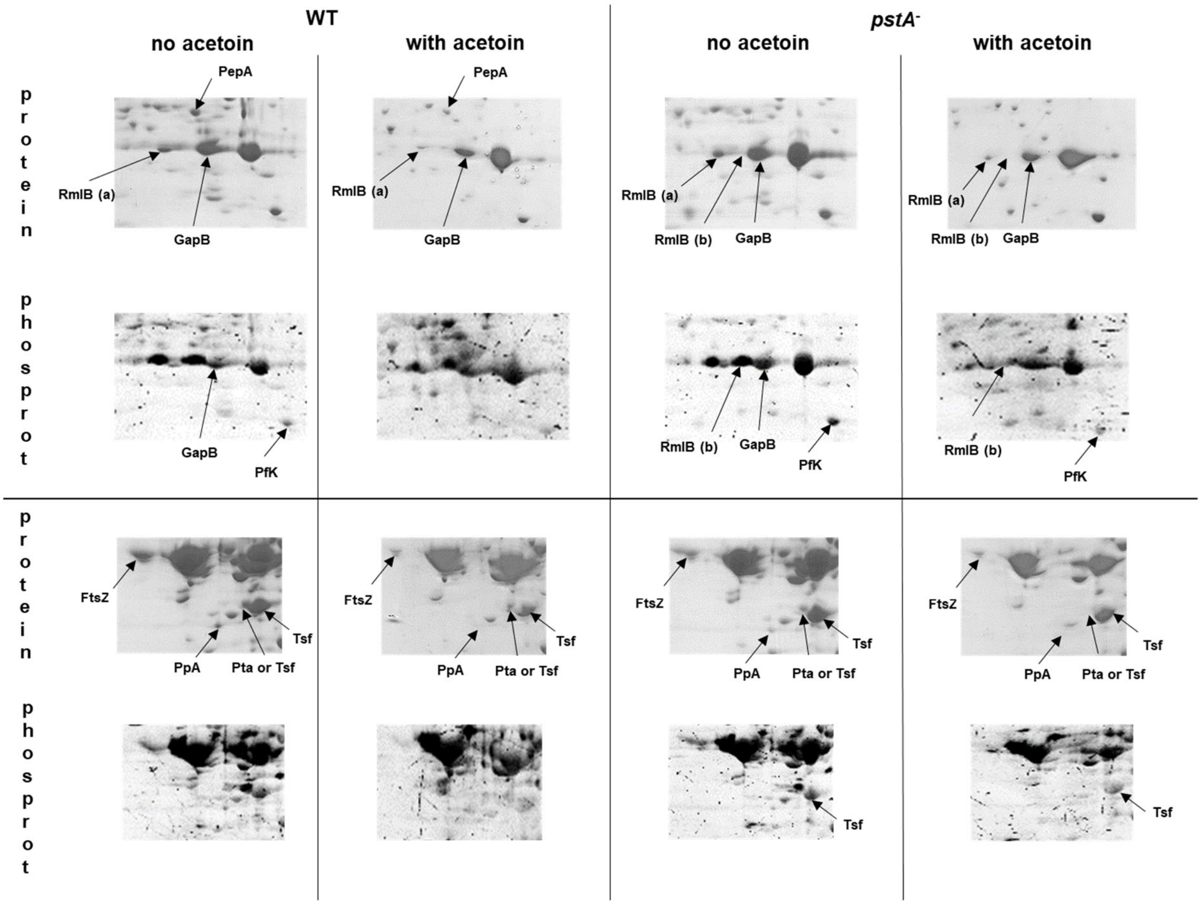
WT
pstA-
no acetoin
with acetoin
no acetoin
with acetoin
protein
phosprot
PepA
RmlB (a)
RmlB (b)
GapB
PfK
FtsZ
PpA
Pta or Tsf
Tsf

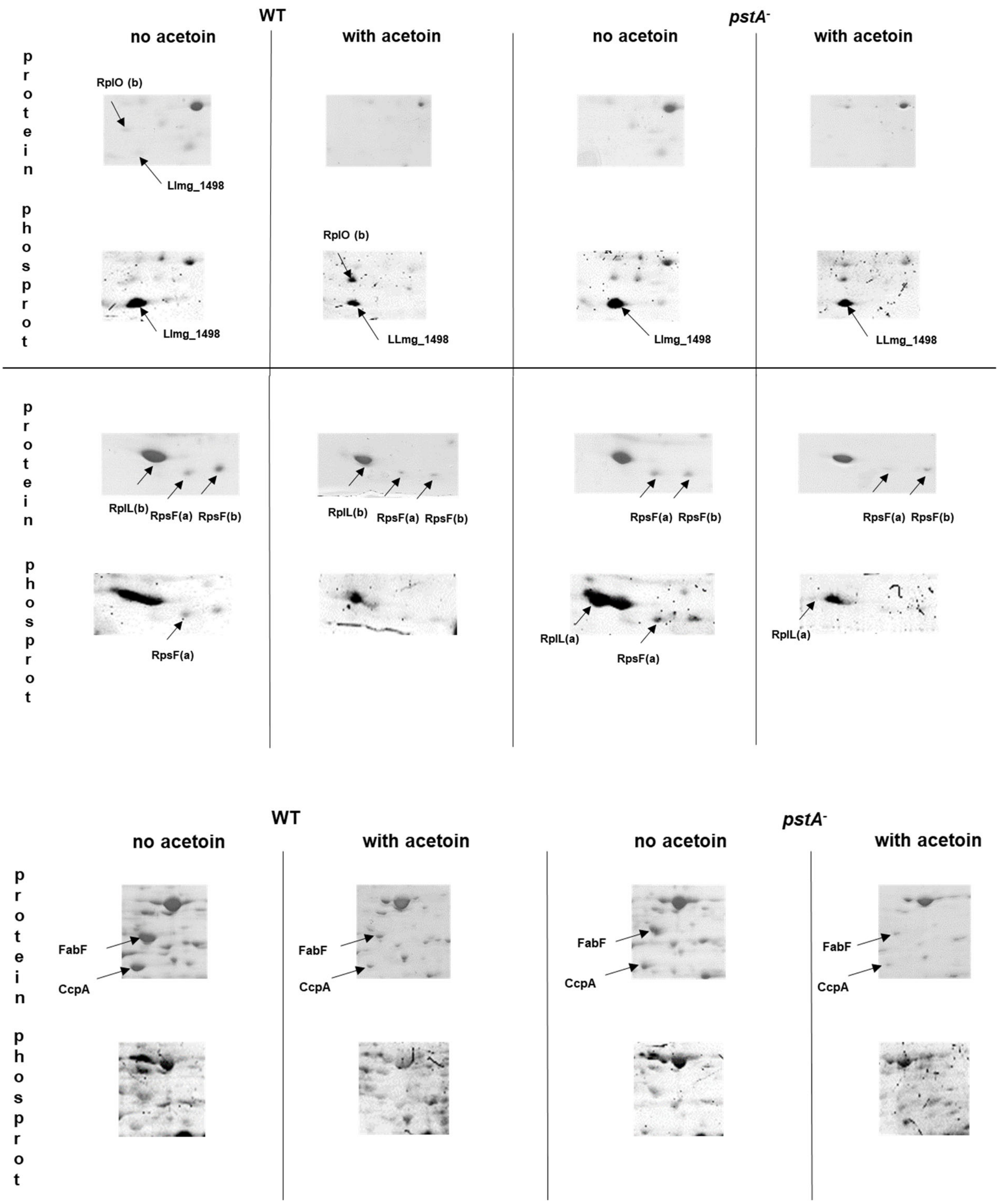

FIG S4. Effects of acetoin in proteome and phosphoproteome of the WT strain and a *pstA* mutant. Cytosolic proteins were separated according to their isoelectric point and molecular weight. Proteins were revealed by Instantblue dye staining and phosphoproteins by ProQ-diamond dye. Figures are representative of two independent experiments.

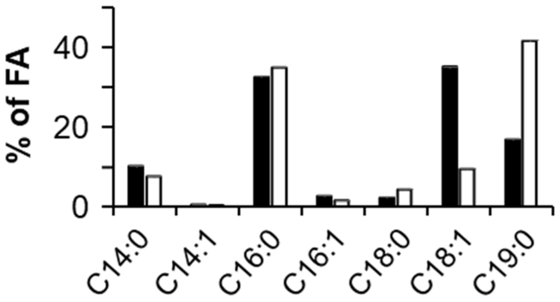

FIG S5. Effect of acetoin on fatty acid (FA) profiles in a *pstA* mutant. The strain was cultured in M17Glu0.5% at 30°C and collected at OD_{600} = 0.5 for membrane fatty acid extraction and analysis. 0.2M acetoin was added at OD_{600} = 0.1. Black bars, no acetoin; grey bars, with acetoin Results are means with standard deviations from three independent experiments.
